# The Role of Cytosolic Lipid Droplets in Hepatitis C Virus Replication, Assembly, and Release

**DOI:** 10.1155/2023/5156601

**Published:** 2023-04-14

**Authors:** Abdullah A. Awadh

**Affiliations:** ^1^Department of Basic Medical Sciences, College of Medicine, King Saud bin Abdulaziz University for Health Sciences, Jeddah 21423, Saudi Arabia; ^2^King Abdullah International Medical Research Center, Jeddah 21423, Saudi Arabia

## Abstract

The hepatitis C virus (HCV) causes chronic hepatitis by establishing a persistent infection. Patients with chronic hepatitis frequently develop hepatic cirrhosis, which can lead to liver cancer—the progressive liver damage results from the host's immune response to the unresolved infection. The HCV replication process, including the entry, replication, assembly, and release stages, while the virus circulates in the bloodstream, it is intricately linked to the host's lipid metabolism, including the dynamic of the cytosolic lipid droplets (cLDs). This review article depicts how this interaction regulates viral cell tropism and aids immune evasion by coining viral particle characteristics. cLDs are intracellular organelles that store most of the cytoplasmic components of neutral lipids and are assumed to play an increasingly important role in the pathophysiology of lipid metabolism and host-virus interactions. cLDs are involved in the replication of several clinically significant viruses, where viruses alter the lipidomic profiles of host cells to improve viral life cycles. cLDs are involved in almost every phase of the HCV life cycle. Indeed, pharmacological modulators of cholesterol synthesis and intracellular trafficking, lipoprotein maturation, and lipid signaling molecules inhibit the assembly of HCV virions. Likewise, small-molecule inhibitors of cLD-regulating proteins inhibit HCV replication. Thus, addressing the molecular architecture of HCV replication will aid in elucidating its pathogenesis and devising preventive interventions that impede persistent infection and prevent disease progression. This is possible via repurposing the available therapeutic agents that alter cLDs metabolism. This review highlights the role of cLD in HCV replication.

## 1. Introduction

Hepatitis C virus (HCV) is a serious public health problem affecting approximately 60 million individuals with chronic HCV infection worldwide [1]. However, most people who encounter the virus often have a persistent infection. HCV infection has been linked to several other illnesses, such as cirrhosis, portal hypertension, hepatic decompensation, and hepatocellular cancer. It is estimated that almost 300,000 people die from the stated health issues each year [2]. Chronic HCV infections induce hepatocellular carcinoma, accounting for over half of all cases in endemic communities. More than 6% of cirrhosis cases worldwide are caused by chronic HCV infections [3]. Perplexing variables that impact HCV infection development include age at the time of infection, insulin resistance, alcohol intake, male gender, obesity, and hepatitis B or HIV coinfections [4].

Lipids are a large group of macromolecules that contribute significantly to cell physiology and disease. Lipids serve three important functions within a cell: a reservoir for neutral lipids, homeostasis of membranes and proteins, and interorganelle crosstalk. The membrane matrix comprises polar lipids, which allow cellular and organelle membranes to fission, fuse, and bud [5]. The role of cLDs in viral replication has been depicted in several clinically important viruses, including HCV, dengue, Zika, herpes, influenza, rotavirus, rabies, and coronavirus. These viruses hijack cLD's metabolism to alter the lipidomic patterns of host cells and therefore enhance their life cycles [6, 7]. Viruses modify the structures, interactions, dynamics, and functions of the cLDs and intracellular lipid species pool [8, 9]. HCV infection induces the accumulation of cLDs in hepatocytes, presumably providing a scaffold for replication and morphogenesis [10]. Likewise, HCV infection induces the upregulation of long fatty acyl chains in triglycerides (TG) and phosphatidylcholines (PC). Higher levels of polyunsaturated fatty acids (PUFAs), including arachidonic acid (AA) (20:4n6), eicosapentaenoic acid (20:5n3), and docosahexaenoic acid (22:6n3), have also been reported upon HCV infection in cultured hepatocytes. De novo lipogenesis is required for efficient HCV replication. Lipid species components of the membrane are also modulated by HCV infection, leading to an altered composition that affects membrane fluidity and curvature. HCV-induced membrane remodeling provides structures for efficient replication and morphogenesis. In recent decades, fatty acids have been shown to perform a range of roles in immunological responses, which include modulation of lipid rafts [11–13], intracellular signaling pathways [14, 15], effects on mitogen-activated protein kinases (MAPKs) [16] and intracellular calcium, interplay with nuclear receptors such as peroxisome proliferator-activated receptors (PPARs) [17, 18], anti-inflammatory eicosanoids implicated in inflammatory response resolution, and impact on a variety of immune cells [15, 19]. There has also been proof of a bidirectional link between the immune system and lipids. Because sterol regulatory element-binding proteins suppress cholesterol production, type I interferon (IFN) signaling activates voluntarily, reducing viral infection [20–22].

Furthermore, following synthesis, the core protein is transported from the endoplasmic reticulum (ER) to the cLDs for temporary storage during HCV replication and then trafficked to the ER to assemble new virus particles [23]. During cell culture infections with the JFH1 (Japanese fulminant hepatitis 1) strain, significant core levels of cLDs have been believed to indicate poor viral generation. The reverse is found in Jc1 (a chimera consisting of J6CF- and JFH1-derived sequences) infections, which are associated with high levels of particle formation [24]. Because the core has been demonstrated to influence cLD accumulation and appearance, this might imply a relationship between viral production control and cLD metabolism [11].

Since the virus demands cellular lipids for multiple critical functions, including replication, particle assembly, and release, the virus's life cycle is extremely reliant on lipid metabolism. HCV collects core proteins and bundles viral genomes via cLDs in the early stages of particle formation. The core and the nonstructural 5A (NS5A) viral proteins increase the biogenesis and accumulation of cLDs in HCV-infected cell cultures [25]. The role of host lipids and lipoproteins in the HCV life cycle has been thoroughly characterized. Lipo-viral particles (LVPs) are complex particles of HCV particles and lipoproteins [26]. Owing to distinct lipoprotein constituents of LVP circulating in the biological milieu, such as cholesterol, TG, and apolipoprotein A, B, E, A-1, and C (ApoB, ApoE, ApoA-I, and ApoC), HCV virus particles can attach to target cell membranes by progressive binding to lipoprotein receptors [27]. HCV then penetrates the cell via clathrin-mediated endocytosis, which includes the involvement of additional host molecules. The HCV particle envelope then fuses with the cellular membrane to release the encapsidated RNA, which uncoats to free the RNA into the cytoplasm to initiate genome translation and replication [28]. Integrating newly synthesized HCV core protein with cLDs is important for HCV genome replication and the formation of progeny viral particles.

HCV makes use of cLDs in a variety of ways to proliferate [29]. Both capsid core and NS5A proteins are localized to cLDs, and their interaction is critical for attracting the replication complex to the cLDs' proximity [29, 30]. Second, in a microtubule-dependent manner, the HCV core stimulates cLD clustering at the microtubule-organizing center, where redistribution is expected to play a pivotal role in effective viral morphogenesis [31]. As an LVP, HCV employs the lipoprotein synthesis mechanism for its assembly and release [32]. These factors can disrupt cellular lipid metabolism, leading to steatosis and associated liver problems. This review is aimed at summarizing recent research on the role of cLDs in the HCV replication cycle, assembly, and, thereby, its release.

## 2. Overview of HCV

**Figure 1:**
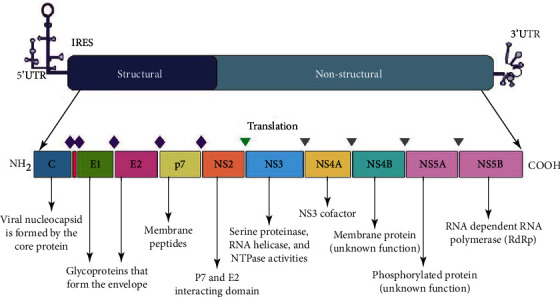
Structure of the HCV genome and translation. The HCV genome comprises a single-stranded positive RNA genome with a single open reading frame that codes for structural and nonstructural proteins and the 5′ untranslated region (UTR) with four highly organized domains, such as I, II, III, and IV, and the IRES. The 3′ UTR consists of stable stem-loop structures and an internal poly(U)/polypyrimidine tract. Depending on the HCV genotype, the single open reading frame codes for a polyprotein with over 3000 amino acids. The 10 HCV proteins' polyprotein enzymatic processing is depicted. The diamond shape (colored purple) represents cleavage sites for host-cell signal peptidase, the triangle shape (colored green) represents autocatalytic cleavage by the NS2-NS3 zinc-dependent metalloproteinase of the NS2-NS3 junction, and the triangle shape (colored grey) represents cleavage sites for the NS3-NS4 serine proteinase complex.

HCV is an enclosed viral particle with a single-strand RNA genome [33]. The strategy starts translation from the HCV genome's open reading frame using the internal ribosome entry site (IRES) to produce a precursor polyprotein for viral proteins [34]. A set of cellular enzymes, such as signal peptidases and viral peptidases, break the precursor polypeptide to release 10 distinct viral proteins [35]. Some proteins undergo significant alterations, such as glycosylation or phosphorylation, to produce biologically developed viral particles. The structure of the HCV genome is presented in Figure 1. The virus particle comprises structural proteins, while nonstructural proteins are only found inside the infected cell and are not formed in the virion. The structural proteins of HCV are the core, envelope-1 (E1), and envelope-2 (E2). The first 191 amino acids of HCV comprise the core, separated into three domains. It is a membrane-bound cytosolic protein associated with hepatocarcinogenesis and steatosis hepatitis, either directly or indirectly [36, 37]. E1 and E2 are two envelope proteins found in HCV. These heavily glycosylated proteins serve a crucial function in cell entry. Cell entry is assumed to be mediated by envelope proteins binding cellular membrane receptor proteins. Each of the remaining nonstructural proteins serves a specific activity. Establishing a viral replication complex, which acts as a step for viral genome replication and mRNA synthesis, is one of the most important roles of the nonstructural proteins. This replication complex is a specific structure generated by the virus infection and protected by a cellular membrane. A nonstructural protein complex is also suggested to play a role in viral particle formation.

NS2 is known to be a viral autoprotease that cleaves NS2 and NS3 to aid viral assembly. NS3 has an N-terminal serine protease and a C-terminal RNA helicase-NTPase. The NS3 protease is important in the processing of HCV polyproteins because it cleaves at four locations downstream of NS3. As a result, NS3 has been a key target in developing HCV antiviral medications. NS4A is an NS3 protease cofactor that forms a stable compound with NS3. NS4B's function is unknown, although it is proven to cause the creation of membranous webs. NS5A is a viral zinc-binding metalloprotein that interacts with various host proteins and signaling cascades. NS5A colocalizes with the HCV core and cLDs and is required for HCV replication [38].

HCV has a substantial genetic diversity and a proclivity for treatment-resistant or immune-evading alterations due to its extremely error-prone activity of RNA polymerase [39]. HCV is divided into nine genotypes, each with a nucleotide sequence that varies by more than 30% [40]. At the 5′ and 3′ ends of the HCV genome, untranslated regions (UTRs) surround one continuous open reading frame. The HCV 5′UTR is divided into four domains, each with well-organized RNA components, such as numerous stem-loops and a pseudoknot 341 nucleotides upstream of the coding area [41, 42]. The 5′UTR contains the IRES. It attracts viral and cellular proteins such as eukaryotic initiation factors (eIF) 2 and 3 to initiate cap-independent HCV genome translation into a single polyprotein [43–45]. The HCV open reading frame contains 9024 to 9111 nucleotides, depending on the genotype. It encodes a single polyprotein, then broken down into 10 unique viral proteins by the host and viral proteases using different characteristics [46].

## 3. Overview of cLDs

**Figure 2:**
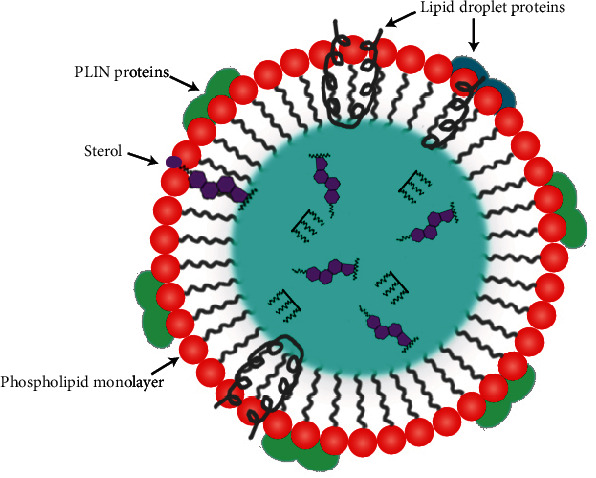
Basic morphology of cLDs. The hydrophobic neutral lipid core of cLDs is encircled by a monolayer phospholipid membrane, which separates hydrophobic neutral lipids from the aqueous cytoplasmic environment. Neutral lipids, primarily TG and CE, are stored in varying ratios in the hydrophobic core of cLDs. Besides membrane phospholipid compositions, the surface protein comprises perilipin (PLIN) proteins.

cLDs refer to the emulsion phase of insoluble oil droplets distributed in the aqueous cytoplasm. The hydrophobic core of cLDs is enveloped by a single layer of phospholipids, distinguishing them from other bilayer cellular organelles (Figure 2). Cholesterol esters (CEs) and TG comprise most of the neutral lipid core [47]. Although the phospholipid monolayer composition varies by cell type, phosphatidylethanolamine (PE) and phosphatidylcholine (PC) are present in all membranes. The cLDs' phospholipid monolayer design significantly impacts their shape and consumption [48]. A rich source of lipolytic enzymes is reported to be found on the surface of the cLDs as a single layer [49]. cLDs are very diverse and present in varying numbers and dimensions, expanding and contracting in response to cellular signals in otherwise identical cells, despite their presence in virtually all cell types across different organisms.

As extensively reviewed, the surface composition of cLDs significantly impacts their size and capacity to interact with other organelles, such as the ER [26, 50, 51]. Specific proteins coat cLDs surfaces, and many of these, predictably, have a role in lipid metabolism. Identification of cLDs proteins in mammalian and yeast cells has been demonstrated in microscopical and nonbiased mass spectrometry analysis studies [50, 52, 53]. The latter method is extremely sensitive, although it is only sometimes precise. Based on these findings, most cLDs have between 50 and 200 distinct proteins on their surface [54]. Protein composition might differ among cLDs of different sizes or other compositions of lipids inside the same cell. cLD proteins targeting signals are discussed elsewhere in [48, 49, 55].

They have diameters ranging from 0.1 to 5 *μ*m in nonadipocytes; however, white adipocytes have sizes exceeding 100 *μ*m. The surface of cLD is bounded by a single layer of phospholipid [56–58]. The presence of free cholesterol (FC) on the surface of cLD is also plausible [59], although the presence of phospholipids may not be ruled out [60]. Lipid esters make up the cLD core. TG predominates in white adipocytes. Although conventional electron microscopy does not reveal any delineation in the core, TG and CE can be distinguished in some cases [61, 62]. Proteins are assumed to be embedded in or adhere to the cLD surface. Furthermore, certain morphological analyses have revealed the existence of proteins in the cLD core, including soluble proteins [63, 64]. This shows that the existing cLD structural paradigm, consisting of a homogenous lipid ester core surrounded by a phospholipid monolayer coated with proteins, can reflect the fundamental properties of cLDs.

### 3.1. Biogenesis of cLDs

The conventional model depicts the fundamental cLD structure by assuming the lipid ester globule is budded and enveloped by the ER membrane [65]. The “hatching” paradigm [66] proposes a bicellular structure and can account for transmembrane ER proteins in segregated cLDs. Nevertheless, biochemical and light microscopic approaches are inadequate to confirm unequivocally that proteins are associated with cLDs rather than related membranes. Another plausible scenario is that lipid esters build up in the membrane of tiny vesicles, obliterating the lumen [67]. The ER-resident diacylglycerol acyltransferases (DGATs) and acyl-CoA:cholesterol acyltransferases (ACATs) catalyze the final steps of TG and CE production, respectively [68, 69]. Though wax esters of bacterial cLDs are anticipated to concentrate on the membrane surface, hydrophobic lipid esters are believed to accumulate inside the ER membrane [70]. When the solubility limit is surpassed, lipid esters are predicted to precipitate between the two sheets. The mentioned state, i.e., lipid ester reserves in the membrane, has only been witnessed in a few cases [70], implying that the structure is challenging to preserve using standard means or that budding cLDs rapidly differentiate from the ER before they become massive enough to be seen microscopically [71]. At least some cLDs are expected to stay linked to the ER. Some proteins migrate back and forth between cLDs and the ER membrane, implying a close structural link [72].

### 3.2. cLD Interaction with Intracellular Organelles

The diverse functions attributed to cLDs mandate regulated communication with nearly all cellular organelles [73]. Due to their monolayer membrane, cLDs form organelle contact sites to facilitate communication by attaching to the organelle surfaces via tether proteins [74, 75]. These contact sites facilitate the exchange of lipids and other components across the contacting organelle to mediate diverse functions, such as signaling, metabolism, organelle biogenesis, and localization [76]. cLD contact sites have been demonstrated to mediate diverse processes in human genetic, infectious, and metabolic diseases. cLD contact sites with several organelles have been shown in several infectious diseases, including viral and intracellular bacteria and parasites. Intracellular pathogens utilize the host cell's lipid species during replication to support energy demand, providing building blocks for pathogen structural components or induction of special replication structures such as those demonstrated in infections with positive-stranded RNA viruses [77, 78].

Poliovirus infection relocalizes cLDs to the replication organelle and subsequently induces contact sites [78]. While the poliovirus proteins 2B and 2C induce cLDs contact with the replication structures, protein 3A mobilizes fatty acids to the replication organelle via interaction with the adipose triglyceride lipase (ATGL) and hormone-sensitive lipase (HSL) [72], and 2A recruits and activates CTP:phosphocholine cytidylyltransferase *α* (CCT*α*) to the replication site [79] to supply lipid for membrane synthesis [80]. These coordinated actions by poliovirus proteins mediate the induction of poliovirus replication structures, presumably protecting the host immune response to support replication and spread [79]. Almost similarly, dengue virus capsid protein facilitates cLDs accumulation near replication compartments, whereby nonstructural protein 4A interacts with cLD-associated ancient ubiquitous protein 1 to induce cLDs contact with autophagosomes to trigger lipophagy [81], liberating lipid species for morphogenesis.

The role of cLDs in HCV replication was elaborated the best among all viruses. RAB18, a small GTPase, facilitates the localization of HCV capsid protein to the surface of cLDs via direct contact with the ER [82]. Along with perilipin 3 (PLIN3), RAB18 mediates cLDs' contact with replication compartments via interaction with HCV NS5A [83]. Overexpression of Rab18 promotes the contact site of cLDs with ER, coupled with a decreased level of PLIN2 on the surface of cLDs [84]. Depletion of PLIN2 alone induces ER wrapping of cLDs and inhibits trafficking of the HCV core to the surface of cLDs [85]. PLIN5 is important in the *β*-oxidation at cLDs by mediating cLD contact with mitochondria. Hence, depletion of PLIN5 abolishes cLDs contact sites with mitochondria and reduces *β*-oxidation [86].

### 3.3. The Role of cLDs in Viral Infections

cLDs are extremely dynamic organelles that change in size and distribution constantly. The availability of sufficient neutral lipid, which is the eventual effect of TG synthesis (lipogenesis) and hydrolysis (lipolysis), and the control of lipolysis by various factors can vary in various cell types, with the size being dependent on the quantity of deposited neutral lipid. Furthermore, cLDs are generally spread throughout the cell and shift in modest oscillations inside a cell; nevertheless, they have been shown to constantly migrate throughout cells during various stress circumstances, such as when HCV infection occurs and cLDs cluster around microtubule organizing centers [31, 87]. cLD levels can also be elevated when cells are infected with HCV and the inflammasome is stimulated. Nod-like receptor protein 3 (NLRP3), an important innate immune mediator, is stimulated when HCV is infected [88]. The inflammasome is activated when NLRP3 is activated [89]. This pathway promotes lipogenic gene upregulation, which may lead to the buildup of cLDs [90].

cLDs are recognized to be used by various viral pathogens to boost infection, highlighting the relevance of cLDs at the host-pathogen interface. At various phases in the life cycle of viruses, linkages between cLDs and +ssRNA viruses, which have close ties with the host ER, are continually forming. West Nile, dengue, Japanese encephalitis, and Zika virus capsid proteins have been found to colocalize with cLDs, and the residues implicated in cLD binding seemed to be conserved [91, 92]. HCV has the most well-studied relationships with cLDs, which are required for morphogenesis and replication [93]. The proximity between cLDs contained in the HCV core protein and the virus assembly complex was assumed to be essential for efficient multiplication and viral assembly during HCV infection. It was shown that higher cLDs during HCV infection are linked to persistent replication and the formation of complexes [94]. Because these replication regions include condensed viral proteins and genetic material, viral proteins may be more likely to be channeled to or interact with cLDs [29–31].

## 4. Role of cLDs in HCV Replication

### 4.1. Role of the Lipid Droplet-Associated Proteins (LDAPs) in the HCV Replication

The core protein has been shown to communicate with a wide range of cellular proteins and impact a wide range of host cell functions [95]. Cell signaling, apoptosis, carcinogenesis, and lipid metabolism are all thought to be implicated by the core protein. PLIN3, also known as tail-interacting protein 47 (TIP47), and PLIN2, also known as adipose differentiation-related protein (ADRP), are the two primary cLD-associated scaffolding proteins present in the liver [96, 97]. PLIN3 is a highly exchangeable protein that has a role in lipid storage, lipid mobilization, and cLD biogenesis, among other aspects [98].

PLIN 2 is a 50-kDa protein found in hepatic cLDs, where it promotes the production of cLDs and protects them against lipolysis, hence mediating lipid buildup [99]. Knocking down PLIN2 reduces lipogenic gene expression, enhances ATGL surface localization, depletes cLDs, and reduces liver TG in high-fat-fed mice by 50%. In macrophages, knocking down PLIN2 enhances lipolysis rate, lowers cLDs per cell by 5.5 times, and lowers cellular TG by 30%. [100]. PLIN2 is a cLD-binding protein rapidly degraded by the ubiquitin/proteasomal pathway after dissociating from cLDs during lipolysis. PLIN2 is being silenced. HCV replication was suppressed following the silencing of PLIN2, and HCV assembly was facilitated. PLIN2 overexpression enhanced the size of cLDs, while PLIN2 silencing decreased them. These findings indicated a novel phenomenon regarding the PLIN2's mechanism of action at multiple stages of the HCV life cycle [101].

PLIN3 or TIP47 is a 47 kDa protein with an initial role in the intracellular trans-Golgi network (TGN) to lysosome trafficking of lysosomal enzymes [102]. PLIN3, unlike PLIN2, is stable in the cytosol and rapidly translocates into cLD synthesis sites after the accumulation of diacylglycerol (DAG) between the ER membrane leaflets upon lipid loading. PLIN3 is undetectable in starved HeLa cells, which lack cLDs before adding fatty acids [98]. In transgenic mice fed either chow or a moderate-fat diet, PLIN3 knockdown reduced HCV core-induced steatosis, which showed that the cLD scaffold protein PLIN3 is required for HCV core-induced hepatic steatosis, which further shed light on the disease's pathophysiology [103]. It has been reported elsewhere that PLIN3 acts as a new cofactor for HCV infection by fusing cLD membranes into the membranous network through its engagement with NS5A [104]. A study demonstrated that PLIN3 mutants that lack the Rab9 binding site misguided newly generated viral particles to the autophagosomal/lysosomal compartment, where they were destroyed. Hence, the Rab9-complexed PLIN3 is essential for optimal HCV particle release [105].

### 4.2. Replication

HCV infection of human hepatocytes is a multistep phenomenon involving various host variables. HCV invasion from the bloodstream into the specific cell depends on viral adhesion to glycosaminoglycans (GAGs) and low-density lipoprotein receptor (LDLR) located on the hepatocyte's basolateral membrane. ApoE promotes HCV entrance by interacting with LDLR and regulates HCV adherence through distinct associations with heparan sulfate proteoglycan (HSPG) [106]. Especially in the initial engagement with GAGs and the LDLR, HCV employs scavenger receptor class B type I (SR-BI), a key high-density lipoprotein (HDL) receptor that also interacts with the very low-density lipoprotein (VLDL) and low-density lipoprotein (LDL) particles in the next phase of infection [107]. The association between SR-B1 and HCV most likely causes the separation of lipoproteins from the exterior of HCV particles. The connection between HCV and the tetraspanin CD81 is required for HCV uptake to commence. By interacting with tight junction proteins, namely, claudin 1 (CLDN1) and occludin (OCLN), the HCV-CD81 complexes cause viral internalization in cholesterol-rich microdomains through a process of clathrin-mediated endocytosis. Endocytosis of HCV in the clathrin vessel causes the merging of viral glycoproteins with early endosomes and vacuole acidification [108]. This mechanism is also aided by ApoC-I, which improves HCV infectivity by interacting with viral glycoproteins [109]. HCV capsid is transported into the cytosol and degraded. The consequent HCV RNA is available for the central element of the replication process following this pH-dependent union between viral and target membranes. A fragmented viral capsid exposes a single-stranded RNA genome with positive polarity to the cytosol following HCV penetration. The HCV RNA is then translated on the rough ER. Both viral transcription and replication processes require the 5′- and 3′-UTR flanking the HCV genome. IRES, positioned in the 5′-UTR of HCV, controls its translation. Attachment to the ribosomal 40S subunit initiates the HCV RNA translation [110].

This process is complemented by the colocalization of nonstructural proteins, which is accomplished by adhering these proteins on membranes that serve as viral replication sites. HCV RNA replication necessitates geranylgeranylated host proteins, as evidenced by the prenylated host FBL2 protein's NS5A binding [111]. It could elucidate the mechanism by which the NS5A protein colocalizes with HCV RNA replication in the membrane. The creation of a unique platform, known as the membranous web (MW), consisting of vesicles and cLDs, is induced by newly formed HCV proteins in combination with host cell proteins [112]. Consequently, HCV nonstructural proteins tweak the ER and cLDs to constitute double-membrane vesicles (DMVs) comprising the complex of HCV assembly [113]. HCV replication assemblies comprise HCV RNA and nonstructural viral proteins [114]. To construct effective HCV replication complexes, significant lipid structural remodeling is required. Aside from structural alterations, HCV infection alters the lipid content of membranes, influencing lipid kinase diffusion in the subcellular space.

**Figure 3:**
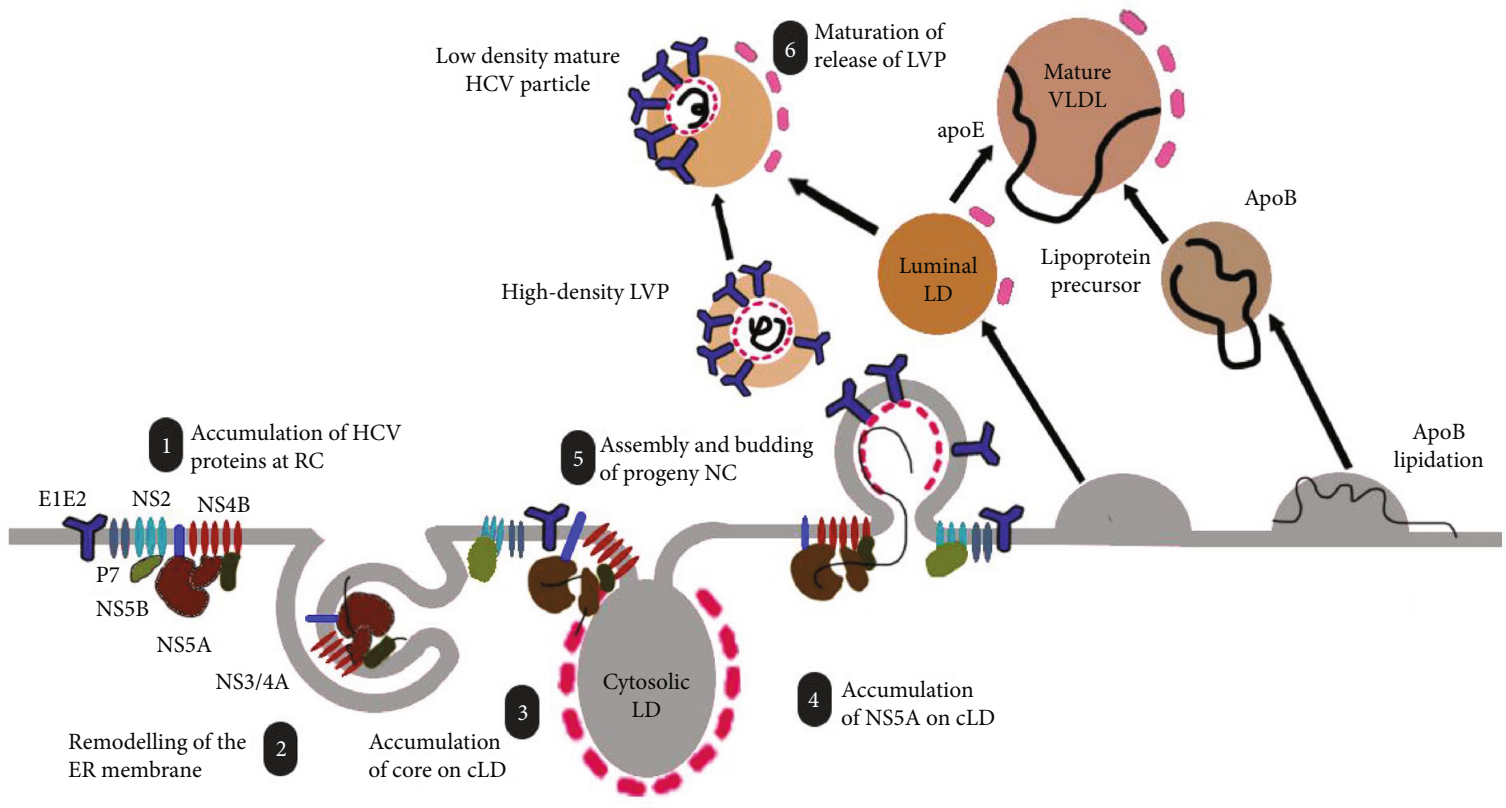
Replication and assembly of viral particles. The replication complex (RC) brings together the NS3 protease and its cofactors, such as NS4A, NS4B, NS5A, and NS5B, to form the “membranous web” (1). NS4B protein mediates intracellular membrane rearrangement, particularly in partially closed double membrane structures where replication occurs (2). Following the assembly of the replication complex, the core accumulates on cLDs which is required to initiate the assembly phase (3). Several endogenous mechanisms coordinate the translocation of NS5A to the LD (4), presumably transferring the nascent HCV RNA to the budding nucleocapsid (NC) (5). Through apoE-E1E2 contact, the immature viral particle merges with or adheres to a luminal lipid droplet for maturation and secretion resembling that of VLDL (6).

This translocation is required to increase the quantity of cholesterol and sphingolipids in membranes. HCV replication has been shown to operate in membranes enriched with cholesterol and sphingolipids [115]. HCV affects the phosphatidylinositol signaling system, causing phosphatidylinositol-4-phosphate to be exchanged with cholesterol in replication compartments. In the HCV RNA multiplication reaction, the positive RNA genome serves as a template for the negative HCV RNA strands. The newly synthesized strands serve as templates for producing positive HCV RNA strands. Novel viral proteins are produced by translating freshly synthesized positive RNA strands. The replication process is represented in Figure 3. The envelope proteins E1 and E2, p7 viroporin, and NS2 protease comprise the NS2 complex. The replication complex combines the NS3 protease and its cofactors, such as NS4A, NS4B, NS5A, and NS5B, to form the MW with the help of several host factors involved in membrane lipid exchange.

### 4.3. Assembly

The proximity of core-enriched cLDs to ER-anchored glycoproteins and replication clusters is most likely required for core depletion, virion assembling, and viral particle generation. The assembly framework does bridge two organelles, the cLDs and the ER, which are connected in a tightly controlled manner. Membrane contact points have recently surfaced as a novel form of interaction [116]. HCV may stimulate membrane interface regions to retain their assembly platform connected, as in-depth reviewed [117].

cLDs are typically found in the cytoplasm of hepatocytes, where they stay stationary or migrate in microtubule-dependent motions. HCV infection or core expression causes cLDs to redistribute to the nucleus periphery over time. The formation of HCV virions requires the core's microtubule-dependent redistribution of cLDs [32]. The relocation of cLDs makes them more accessible to replication complexes, where most NS proteins are found and HCV RNA is replicated. Mutations that restrict core binding to cLDs impede cLDs from being close to RNA synthesis sites. It indicates that the core (on the surface of cLDs) interacts with NS5A (on the ER near the replication vesicles) to enable the transfer of RNA for packaging into assembling virions. The basic motif seen in the core and NS5A is required for interaction with HCV RNA [118]. Because they may compete with RNA-replication proteins, core, and NS5A do not localize to replication complexes preceding HCV RNA replication. At the start of the assembly, the HCV core and, to a lesser extent, NS5A relocate to the cLDs surface. A group of researchers reported that DGAT1 binds to both the core and NS5A, suggesting that DGAT1 stabilizes the core-NS5A connection on the surface of cLDs [119]. Although accumulation of cLDs has not been shown to correlate with HCV replication across strains and genotypes [120], the cLDs in HCV replication extend beyond the only accumulation, as demonstrated previously with the Jc1 strain which replicates to high titer without inducing accumulation of clustering of enlarged cLDs, but small and scattered through the cytoplasm [121]. cLDs are laboriously sensitive for manipulation in cell culture. Subculturing, reseeding, or changing culture density would easily alter their intracellular dynamics. Likewise, several strains/genotypes have different spatiotemporal dynamics of cLDs across the time after infections and culture density [122]. Further, automated analysis of cLDs (e.g., 3D reconstruction) may not be straightforward due to the sensitivity and quick photobleachability of the fluorochrome Bodiby [123]. This is particularly problematic in detecting small cLDs, allowing detection and enumeration of only enlarged cLDs, which vary greatly in response to biological manipulation such as infection where the rate of fusion into enlarged cLDs or division into smaller ones is hardly detectable in imaging of fixed cells [124].

ApoE, ApoB, ApoA1, and ApoC1 are all involved in producing HCV virions [125]. The microsomal triglyceride transfer protein (MTP) that facilitates the transfer of neutral lipids from cLDs to building ApoB-containing particles has also been linked to HCV-induced steatosis. ApoE is the most important apolipoprotein for HCV assembly, as genetic reduction of ApoE prevents the virus from forming [126]. Although the level of virion ApoB is linked to infectivity, the usage of Huh-derived cell lines may undervalue ApoB's importance.

### 4.4. Release

**Figure 4:**
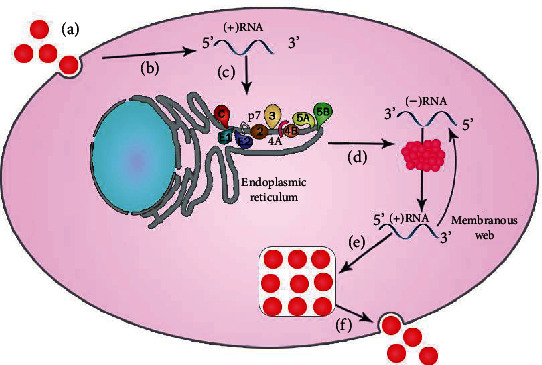
Lifecycle of HCV. (a) Binding of virus along with its internalization. (b) Release in cytoplasm followed by uncoating. (c) IRES-mediated translation and processing of polyprotein. (d) RNA replication. (e) Assembly of virions. (f) Maturation of virions and their subsequent release.

Generally, HCV virions are assumed to mature and release via the same mechanism as VLDLs, which transport cholesterol and TGs from hepatocytes [127]. Virus particles in infected persons are crosslinked with VLDLs and are referred to as LVPs [27]. LVPs contain viral RNA, core protein, and the VLDL structural elements ApoB and ApoE, as well as viral RNA and core protein [27, 128]. The specific lifecycle of HCV is presented in Figure 4. Several investigations have found that viral particle release is influenced by VLDL synthesis and secretion. However, evidence also suggests that ApoE is required for assembly and, thereby, the release of the virus. At the same time, levels of ApoB and the function of MTP function have a negligible or minimal effect [129, 130]. The study found that small interfering RNAs targeting ApoE lowered intrinsic and extrinsic infectious titers, but those aiming at ApoB had no influence. Inhibitors of MTP also blocked ApoB production but not ApoE/virus discharge, albeit higher inhibitor concentrations led to a decline in virus concentration, replicating the effects of ApoE secretion delays [130]. Previous findings that implicated ApoB and MTP in HCV assembly and release may have been influenced by toxicity and high inhibitor doses. Thus, while there is unanimity that the assembly of VLDL is required for the release of HCV, the function of specific variables in VLDL generation is still a point of contention.

According to research findings, processes driving endosome development have recently been highlighted as another cellular pathway implicated in particle escape [131]. Endosomes are formed by multiprotein complexes known as the endosomal sorting complexes required for transport (ESCRT). These work by integrating target proteins into a multivesicular body for destruction [132]. Following that, ESCRTs are dissociated from membranes which are ultimately recycled for consecutive rounds of vesicle synthesis [133]. When the vacuolar protein sorting-associated protein 4 (Vps4) function is disrupted, ESCRTs become defective, and abnormal endosomes develop [134]. Surprisingly, the expression of dominant-negative ESCRT components and Vps4 lowers HCV particle production. These findings show that ESCRT has a role in the viral escape, while the exact role of endosomal complexes is unknown. HCV can now be transmitted from infected to uninfected ones, according to new research endeavors [135]. Inoculating monolayers at a denser population, which allows close cell communication, aids this process. Even though the whole complement of viral proteins, including the E1 and E2 envelope glycoproteins, is required for cell-cell transmission, the properties of virus particles that facilitate cellular transmission are still unknown [136].

## 5. Practical Insight into an Infectious HCV Particle

When HCV in patients' blood is evaluated, the virus appears as a fragmented colony with varying densities. HCV has been linked to an array of lipoproteins, which have a remarkable role in its replication and assembly [137]. LDL receptors on cell cultures were assumed to be important in virus endocytosis, including HCV and bovine viral diarrhea virus (belonging to the family Flaviviridae) [138]. It has been demonstrated that the interplay with VLDL is critical for virus particle production and pathogenicity accumulation [139]. VLDL is a lipid particle that contains apolipoproteins and has a hydrophobic neutral lipid core surrounded by a single amphipathic layer of phospholipid and unbound cholesterol [140]. The precursor of VLDL is most likely accumulated in the ER lumen's microenvironment, where core-layered cLDs interface with ER membranes enriched in replication complex and nonstructural proteins complex, raising the number of antecedent VLDL and permitting it to engage with HCV, which is also reattached to the ER lumen. The revelation that an inhibitor of MTP reduces the generation of infectious virus particles suggests that lipoproteins are important for HCV infectivity [141]. Considering the suppression of pathogenic viruses released into growth media, intracellular infectious virus particles in cells exposed to MTP showed a slightly greater density than those released into growth media. Another reason for the MTP inhibitor's findings is that it can alter the formation or functionality of another cellular component (s) that interact with HCV or regulate pathogenic HCV production. Furthermore, it is claimed that ApoE and ApoB are significant host components associated with endowing infectivity to HCV, at least to some degree. It has been demonstrated that the engagement of core-enveloped cLDs with the ER membrane is critical for producing pathogenic viral particles. The affiliation with HCV, which is also regrown into the ER lumen, may be facilitated by a higher concentration of VLDL precursor. Because NS5A binds to ApoB20 and ApoE, it is plausible that the NS proteins help VLDL engage with HCV. Although VLDL has been linked to the development of infectious HCV, it is unclear if VLDL interacts with the virus before it is released into the culture media.

HCV infection is thought to be regulated through several cellular receptors related to lipid metabolism, namely, the LDLR and SR-BI at the basolateral surface and the Niemann-Pick C1-like 1 (NPC1L1) at the apical surface will be briefly explained here. Comprehensive coverage of all host receptors involved in HCV entry has been discussed elsewhere [142, 143]. Approximately a decade after the discovery of HCV, the role of LDLR in HCV entry has been reported [144]. Competition experiments involving serum HCV particles and human lipoproteins demonstrated that LDL and VLDL inhibited HCV binding and entry into cells, indicating that LDLR is involved in uptaking viral particles [138]. The same has been reproduced with HCVcc and hepatoma cells (Huh-7) [145] as well as in a more physiologically authentic cell culture model, such as the primary human hepatocytes (PHH) infection by serum HCV [146]. Likewise, recombinant soluble peptides or monoclonal antibodies against LDLR inhibited HCV entry into PHH. Furthermore, LDLR knockdown reduced HCV infection of hepatoma cells, which was restorable upon ectopic expression [147]. SR-BI is a receptor for high-density lipoproteins (HDLs) [148]. SR-BI's role in HCV entry was demonstrated by its interaction with a soluble fragment of HCV envelope E2 [149]. Antibody against SR-BI soluble E2 inhibited sE2 binding to the cell surface [150], while ectopic expression of SR-BI on nonpermissive cells for HCV allowed binding of HCV sE2 [151]. Similar results were shown in PHH [152] and immunosuppressed chimeric mice with humanized livers [153]. NPC1L1 is a receptor for the absorption of cholesterol that was also shown to involve in HCV entry. Silencing NPC1L1 or blocking by an antibody led to a drastic reduction in HCV infectivity. The NPC1L1 small molecule inhibitor, ezetimibe, inhibits HCV entry in cells and mice with humanized livers. It was hypothesized that NPC1L1 facilitates the last step of binding and recognition of HCV [154].

**Figure 5:**
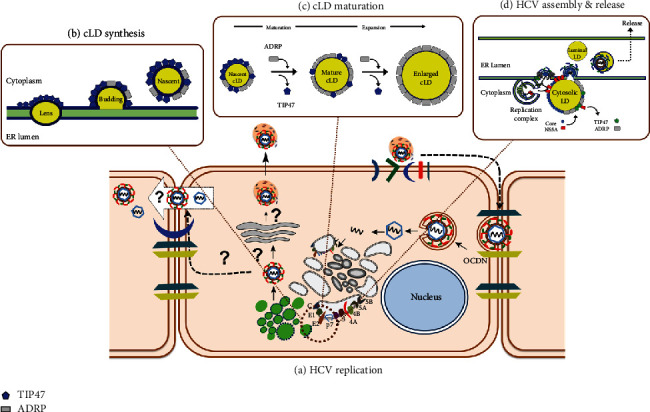
Coupling of cLD metabolism and HCV replication. (a) HCV replication induces extensive ER membrane remodeling to establish a structural complex for replication and morphogenesis. (b) cLD-associated proteins (LDAPs), particularly TIP47 and ADRP, are essential in initiating the morphogenesis of cLDs, initially by coordinating the accumulation of neutral lipids at the ER leaflets and eventually the excision of the cLD from the ER. (c) LDAPs, including TIP47 and ADRP, regulate the metabolic dynamic of cLDs in response to the metabolic status of the cell as to whether they stimulate lipogenesis (cLD enlargement) or lipolysis (cLD diminution). (d) The HCV assembly and release are coupled with the metabolic and structural dynamics of cLDs and apolipoproteins. LSAPs like ADRB and TIP47 mutually coordinate the metabolism and morphogenesis of cLDs and directly interact with HCV proteins like core and NS5A to facilitate the assembly and release of HCV progeny particles.

In summary, HCV replication and morphogenesis characterization revealed an interdependent and intricate interaction with cLD metabolism and morphogenesis [155]. HCV replication induces extensive ER membrane remodeling to establish a structural complex for replication, which is adjacent to clustered cLDs where HCV morphogenesis is thought to occur [156]. The HDV-induced remodeling of ER results from a coordinated interaction between the HCV proteins, notably NS5A and NS4B, integral LDAPs such as ADRP and TIP47, phospholipid metabolizing enzymes, and proteins mediating membrane fusion and excision [105, 157, 158]. TIP47 and ADRP coordinate the initiation of the cLD morphogenesis by recruiting and stabilizing the enzymes that accumulate neutral lipids at the ER leaflets until reaching the critical size for excision into the cytoplasm [159]. In hepatocytes, TIP47 and ADRP primarily regulate the metabolic dynamic of cLDs in response to the metabolic status of the cell via the liberation of fatty acids from cLDs for b-oxidation (lipolysis) when the cell's energy demand increases or re-esterification of free fatty acids into TG during absorption of excess fatty acids (lipogenesis) [160]. The HCV assembly and release steps are the least characterized, mostly because of the unauthentic HCV species that replicate efficiently in cell culture to enable microscopic or biochemical characterizations. The subcellular HCV-induced membranous structures and interacting organelles such as cLDs are below the resolution limit of most common high-resolution microscopic systems and attempts to visualize HCV particles with electron microscopy had yet to produce conclusive results [161, 162]. However, biochemical and genetic methods demonstrated tight coupling of the HCV assembly and releases and cLD morphogenic and metabolic dynamics (Figure 5).

## 6. Conclusion

The current review draws on our knowledge of cLD cellular biology and liver lipoprotein production to elucidate the framework by which HCV creates its LVPs using these stated organelles and pathways. The current article focuses on the frameworks of viral protein dispersal onto the cLDs, as well as the coordination of functionality between the processes of replication and assembly, the importance of TG mobilization from cLDs for HCV arrangement, the engagement between HCV and the lipoprotein synthesis evolution, the role of apolipoproteins in virion assembly, and the ramifications of these findings.

The paucity of a comprehensive understanding of the VLDL secretion cascade is a fundamental roadblock in comprehending HCV assembly. While lipoproteins are a major element of plasma and metabolism in terms of quantity and functionality, there are currently only models known to elucidate their generation. To complete the vision, further imaging data, precise purification processes, and cellular systems reproducing liver features as precisely as feasible are sought. The collaboration of cell biologists and virologists will be critical in furthering our knowledge of the host machinery that supports HCV development. The vast quantity of data gained in recent years on the role of lipid metabolism in the replication and assembly of HCV and its influence on virion properties will aid in vaccine development chances as we gain a better understanding of hepatic lipid metabolism processes.

## Data Availability

No datasets were analyzed or generated during the construction of the submitted manuscript.
